# Spred2 Modulates the Erythroid Differentiation Induced by Imatinib in Chronic Myeloid Leukemia Cells

**DOI:** 10.1371/journal.pone.0117573

**Published:** 2015-02-17

**Authors:** Yuefeng Yang, Xiaoyun Liu, Fengjun Xiao, Shuya Xue, Qinqin Xu, Yue Yin, Huiyan Sun, Jie Xu, Hengxiang Wang, Qunwei Zhang, Hua Wang, Lisheng Wang

**Affiliations:** 1 Department of Experimental Hematology, Beijing Institute of Radiation Medicine, Beijing, PR China; 2 Center for Disease Control and Prevention of Lanzhou Command, Lanzhou, PR China; 3 Department of Hematology, Peking University First Hospital, Beijing, PR China; 4 Department of Hematology, General Hospital of Air Force, Beijing, PR China; 5 Department of Oncology, Qinghai Provincial People’s Hospital, Xining, PR China; Sun Yat-sen University, CHINA

## Abstract

Differentiation induction is currently considered as an alternative strategy for treating chronic myelogenous leukemia (CML). Our previous work has demonstrated that Sprouty-related EVH1 domainprotein2 (Spred2) was involved in imatinib mediated cytotoxicity in CML cells. However, its roles in growth and lineage differentiation of CML cells remain unknown. In this study, we found that CML CD34^+^ cells expressed lower level of Spred2 compared with normal hematopoietic progenitor cells, and adenovirus mediated restoration of Spred2 promoted the erythroid differentiation of CML cells. Imatinib could induce Spred2 expression and enhance erythroid differentiation in K562 cells. However, the imatinib induced erythroid differentiation could be blocked by Spred2 silence using lentiviral vector PLKO.1-shSpred2. Spred2 interference activated phosphorylated-ERK (p-ERK) and inhibited erythroid differentiation, while ERK inhibitor, PD98059, could restore the erythroid differentiation, suggesting Spred2 regulated the erythroid differentiation partly through ERK signaling. Furthermore, Spred2 interference partly restored p-ERK level leading to inhibition of erythroid differentiation in imatinib treated K562 cells. In conclusion, Spred2 was involved in erythroid differentiation of CML cells and participated in imatinib induced erythroid differentiation partly through ERK signaling.

## Introduction

Chronic myelogenous leukemia (CML) arises mostly from a pluripotent hematopoietic stem cell that contains thereciprocal t(9;22)(q34;q11) chromosomal translocation coding BCR/ABL fusion oncoprotein. BCR/ABL kinase activates a variety of downstream survival pathways and inhibits cell differentiation [1,2]. The CML is currently successfully treated with BCR-ABL inhibitors, such as imatinib and dasatinib [3–5]. However, clinical resistance to these drugs has also been widely reported in CML patients [6–9].

CML is a clonal hematopoietic stem cell disorder that the malignant clone progressively loses the capacity for terminal differentiation. Thus, differentiation induction has been considered as an alternative approach for CML therapy. Some valuable progress has been achieved in biological or chemical agents that could induce terminal differentiation [10–13]. It has been reported that low concentration of imatinib induces proliferation arrest and erythroid differentiation of CML cells [14,15]. The RAS-ERK pathway is known to contribute to myeloid differentiation of CML cells [16]. Notably, CML treatment lead to terminal differentiation of leukemia cell lines or primary cells, as well as proliferation arrest and cell apoptosis, by regulating RAS-ERK cascade [17–20].

Sprouty-related EVH1 domainprotein 2 (Spred2) proteins are identified as a family of membrane-associated negative regulators of growth factor-induced RAS-ERK activation [21]. Our previous studies demonstrated that Spred2, a subset of Spreds family, was involved in imatinib-induced cytotoxicity in CML cells. Imatinib treatment upregulates Spred2 expression, leading to apoptosis and growth arrest in CML cells [17]. However, whether Spred2 is implicated in CML cell differentiation remains unclear. In this study, we clarified the expression and potential roles of Spred2 protein in erythroid differentiation of CML cells and its mechanisms.

## Methods

### Cell lines and primary cells

The human myelogenous leukaemia cell line K562 were obtained from America Type Culture Collection (ATCC, Manassas, VA, USA) and cultured in RPMI-1640 (Sigma, St. Louis, MO, USA) medium containing 10% heat-inactivated fetal calf serum (FCS, Hyclone, Logan, UT, USA), 100 unit/ml penicillin and 100 μg/ml streptomycin in a humidified 5% CO_2_ atmosphere at 37°C.

The bone marrow (BM) samples were obtained from healthy donor or CML patients undergoing diagnostic procedures at Peking university first hospital. Written informed consent was obtained from each healthy donor and CML patient. All the procedures were approved by the Ethics Committee of Beijing Institute of Radiation Medicine. Mononuclear cells were isolated from heparinized samples by centrifugation through a Ficoll-Hypaque density gradient (Amersham Biosciences, Piscataway, NJ, USA). Then, CD34^+^ cells were isolated by using human CD34 positive selection kit (Stem Cell Technology, Vancouver BC, Canada).

### Lentiviral vector production

Lentiviral shRNA vector targeting Spred2 (PLKO.1-shSpred2) was constructed according to the protocol of PLKO.1-puro vector (Addgene, Cambridge, MA). Briefly, the forward oligo, 5’ccggtggtattggaatgctatgtaactcgagttacatagcattccaataccatttttg 3’ and reverse oligo, 5’aattcaaaaatggtattggaatgctatgtaactcgagttacatagcattccaatacca3’ were annealed and inserted into the PLKO.1-puro vector, which was digested by AgeI and EcoRI. And, control vector PLKO.1-shScramble was also purchased from addgene. Then, the 1406 bp fragment between XbaI and BamHI was obtained from plasmid pHIV7-SF-RFP, and cloned into the corresponding sites (SpeI and BamHI) of PLKO.1-shSpred2 or PLKO.1-shScramble, respectively.

293T cells (ATCC) were cultured in RPMI 1640 (Sigma) medium supplement with 10% FCS (Hyclone) and plated at 6×10^6^ cells per 10-cm plate 1 day before transfection. Transfer vector PLKO.1-shSpred2 or PLKO.1-shScramble, packing plasmid psPAX2 and envelope plasmid pMD2.G were co-transfected by using the phosphate coprecipitation kit (Promega, Madison, WI, USA) according to manufacturer’s protocol and culture medium was replaced by fresh growth medium 6h after transfection. The virus containing media were collected at 36h and 48h after transfection. Viruses were purified and concentrated by PEG, followed by determination of viral titers on HT1080 cells.

### Virus transduction

Before transduction, CD34^+^ cells were cultured in SFEM medium (Stem Cell Technologies. Inc., Vancouver, Canada) supplement with 50ng/ml stem cell factor (SCF), 100ng/ml thrombopoietin (TPO), 100ng/ml FMA-like tyrosine kinase 3 ligand (Flt-3L), 100 ng/ml interleukin (IL) -6, and 50ng/ml IL-3 (Peprotech, Rocky Hill, NJ) for 48 hours. CD34^+^ cells and K562 cells were plated in 24-well plate at a density of 2×10^5^ per well, and then were infected by lentiviral vectors at multiplicity of infection (MOI) of 10 or by adenoviral vector at MOI of 150. The gene transduction efficiency of lentiviral vectors, indicated by RFP expression, was detected by flow cytometry (Becton Dickinson, Mountain View, CA).

### Differentiation assay

For differentiation assay, CD34^+^ cells infected by viruses were cultured in Iscove’s Modified Dulbecco Medium (IMDM) supplement with 30% FCS, 50 ng/ml SCF, 50 ng/ml IL-3, 200 ng/ml granulocyte colony-stimulating factor (G-CSF), 200 ng/ml granulocyte-macrophage colony stimulating factor (GM-CSF) (Peprotech), 63 μM β-mercaptoethanol and 3 unit/ml erythropoietin. At day 0, 3 and 7 post-infection, cells were collected and labeled with allophycocyanin (APC)-conjugated anti-human CD34 antibody, fluorescein isothiocyanate (FITC)-conjugated anti-human CD235a antibody and Phycoerythrin (PE) Cy7 conjugated anti-human CD11b antibody (BD Biosciences, San Jose, CA), and then detected by flow cytometer.

The differentiation of K562 cells transduced by lentivrial vectors or adenoviral vectors was induced by 10ng/ml Phorbol-12-myristate-13-acetate (PMA, Sigma Chemical Co., St. Louis, MO) or 1μM imatinib (sigma), respectively. At indicated time points, cells were collected and labelled with PE/FITC-conjugated CD235a antibody, and then detected by flow cytometer.

And, the mRNA expression of Spred2, CD235a and differentiation related transcription factors GATA1 were also detected in CD34^+^ cells and K562 cells by using real-time reverse transcription polymerase chain reaction (RT-PCR).

### Colony-Forming Cell (CFC) assay

Two days after transduced by lentiviral vectors, CD34^+^ cells were plated in 24-well plate at a density of 500 per well, and cultured in 1% methylcellulose medium supplemented with 30% FCS, 50ng/ml SCF, 50ng/ml IL-3, 200ng/ml G-CSF, 200ng/ml GM-CSF, 63μM β-mercaptoethanol and 3 unit/ml erythropoietin, which is formulated to support optimal growth of erythroid progenitors (CFU-E and BFU-E), granulocyte-macrophage progenitors (CFU-GM, CFU-G, and CFU-M) and multi-potential granulocyte, erythroid macrophage and megakaryocyte progenitor (CFU-GEMM). Fourteen days later, the presence of colonies (>40 cells) was counted and scored. The colonies formation scoring and erythroid colonies scoring were calculated from the numbers of colonies/total number of cells seeded.

### Real-time RT-PCR

Total RNA was isolated from CD34^+^ cells or K562 cells by using TRIzol reagent (Invitrogen, Carlsbad, CA), and the cDNA was synthesized using a First Strand cDNA Synthesis Kit (Thermo Scientific, Wilmington, DE) according to the manufacturer’s instructions. Then, the mRNA expression was quantified by using SYBR Green Real-Time kit (Takara Bio Inc., Otsu, Shiga, Japan) on 7500 Fast Real-Time PCR System (Applied Biosystems, Foster City, CA). The primers for homo sapiens CD235a, spred1, spred2, gata binding protein 1 (globin transcription factor 1) (GATA1) and beta-actin (β-actin) were shown in Table 1. And the expression levels were normalized by β-actin or the target gene expression at day 0 after cultured in differentiation medium. The results were showed as the mean± s.d. of triplicates and were representative of three independent experiments.

**Table 1:** Primers for real-time PCR detection.

genes	Sequence
CD235a	Sense: 5’-aagggtacaacttgcccatca-3’
	Antisense: 5’-ttcaacagaacttaaaggcacgtc-3’
Spred1	Sense: 5’-ggaagcactagaaactggcattatt-3’
	Antisense: 5’-cacctggctgctaggcaaac-3’
Spred2	Sense: 5’-ctcatccatggtgaacgacagaa-3’
	Antisense: 5’-tgtcaaaggctcgggcatc-3’
GATA1	Sense: 5’-ctgcggcctctatcacaagatg-3’
	Antisense: 5’-actgagtacctgcccgtttactgac3’
PU.1	Sense: 5’-tgaaggacagc atctggtg-3’
	Antisense: 5’-ccgtcttgccgtagttgc-3’
β-actin	Sense: 5’-gcgggaaatcgtgcgtgac-3’
	Antisense: 5’-ggaaggaaggctggaagag-3’

### Western blotting

After indicated treatment, Spred2 over-expressed or slicenced K562 cells were collected and the protein was extracted. Then, the expression of Spred2 was detected by rabbit anti-human Spred2 antibody (Sigma). And, the activation of MAPK signalling pathway was detected by anti-phospho-ERK1/2 antibody and anti-ERK-1/2 antibody (Santa Cruz Biotechnology, Santa Cruz, CA) at 6h, 12h, 18h, 24h after treatment with 10mg/ml PMA or at 1h after treated by 0.1, 0.5 or 1.0μM imatinib.

### Statistical analysis

All results are representative of at least three independent experiments. Values were presented as the mean ± SD. One-way analysis of variance was used to compare the means of two or more experimental groups, followed by the Dunnett post hoc test. The difference was considered to be statistically significant as p<0.05.

## Results

### Spred2 induced erythroid differentiation of NBM CD34^+^ cells

**Fig 1:**
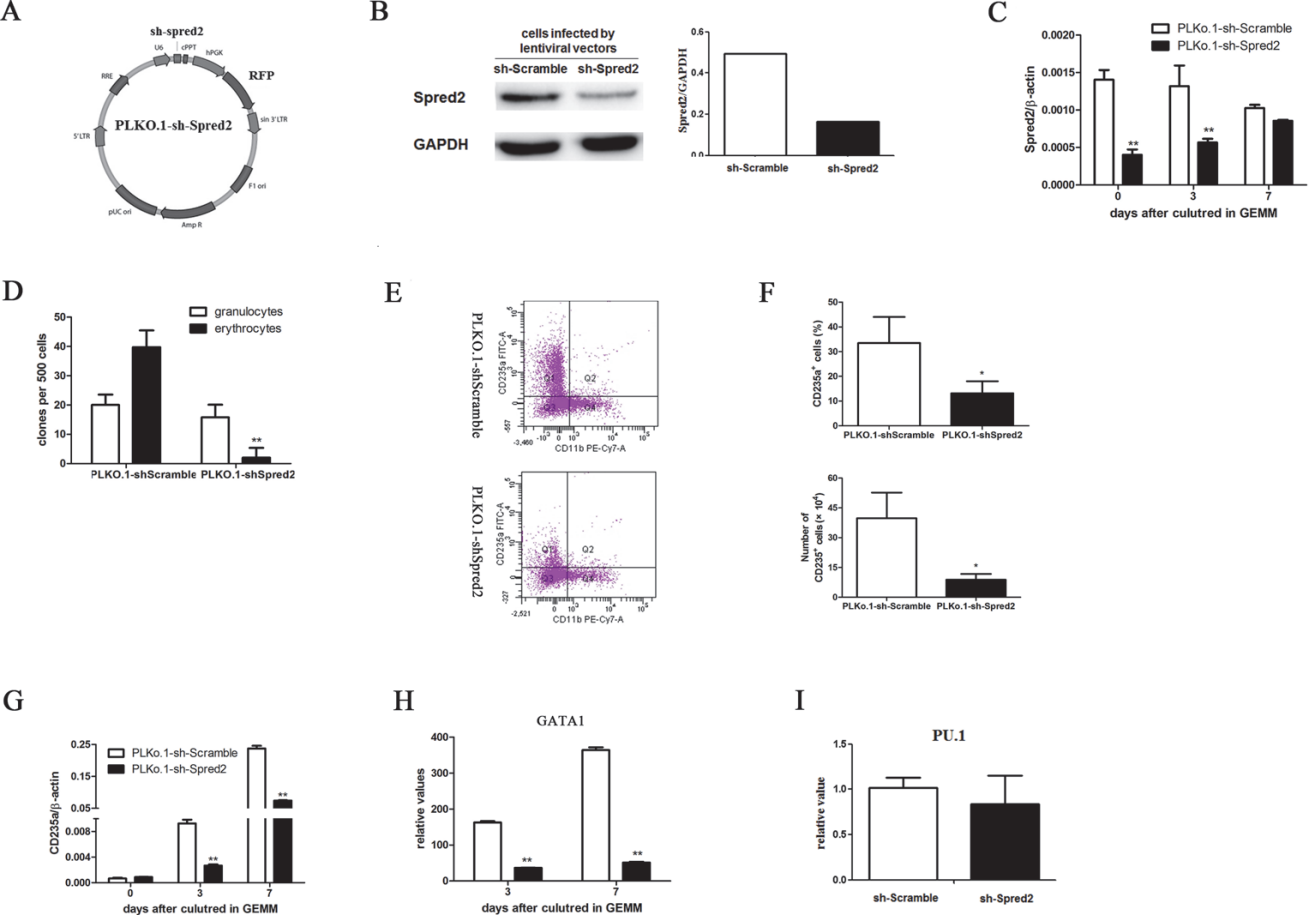
Spred2 interference suppressed the eryhtroid differentiation of human normal bone marrow (NBM) CD34^+^ cells. The scheme of lentivirus vector with shRNA specifically targeting Spred2, PLKO.1-shSpred2, was shown in A. 48h after transduced with 5 multiplicity of infection (MOI) PLKO.1-shSpred2 or PLKO.1-shScramble, the expression of Spred2 was detected to confirm the interference efficiency by Western-blotting (B) in human NBM CD34^+^ cells. Human NBM CD34^+^ cells were infected by PLKO.1-sh-Scramble or PLKO.1-sh-Spred2 at a MOI of 10 and cultured in GEMM medium. At day 0, 3 and 7 post-infection, the expression Spred2 was confirmed by real-time PCR (C), and the expression of CD34, CD235a and CD11b were analyzed by flow cytometer (E-F). Furthermore, the mRNA expression of CD235a (G), GATA1 (H) and PU.1 were detected by real-time RT-PCR. The expression of GATA1 expression was normalized by the expression level at day 0. And, the PU.1 expression at day 3 post-infection was normalized by the expression in PLKO.1-sh-Scramble group (I). PLKO.1-sh-Scramble or PLKO.1-sh-Spred2 transduced CD34^+^ cells were plated in 24-well plateand cultured in GEMM medium plus 1% methylcellulose for 14 days the presence of colonies (>40 cells) was counted and scored (D). Data are mean±s.d. of three independent experiments. *, p<0.05; **, p<0.01 vs the PLKO.1-sh-Scramble group at the same time point.

To clarify the roles of Spred2 in erythroid differentiation of normal hematopoietic stem/progenitor cells, the NBM CD34^+^ cells were transduced with PLKO.1-shSpred2, a lentivirus vector with shRNA specifically targeting Spred2 (Fig. 1A–1B), or PLKO.1-shScramble. Spred2 expression was downregulated during the differentiation in PLKO.1-shScramble transduced NBM CD34^+^ cells, while PLKO.1-shSpred2 stably silenced Spred2 expression until 7 days after cultured in GEMM medium (Fig. 1C). Our data showed that Spred2 interference inhibited the CFU-E (erythroid colony-forming units) obviously, but not CFU-G (granulocyte colony-forming units) (Fig. 1D). Moreover, CD235a expression could be induced by GEMM differentiation medium in NBM CD34^+^ cells, while Spred2 silence reduced the CD235a expression obviously (Fig. 1E–1G). Our results also showed that erythroid related transcription factor GATA1 was downregulated by Spred2 silence (Fig. 1H). However, Spred2 interference had little effect on the expression of transcription factor PU.1, which supports myeloid cell lineage differentiation, at day 3 post-infection (Fig. 1I).

### Spred2 restoration promoted erythroid differentiation of CML CD34^+^ cells

**Fig 2:**
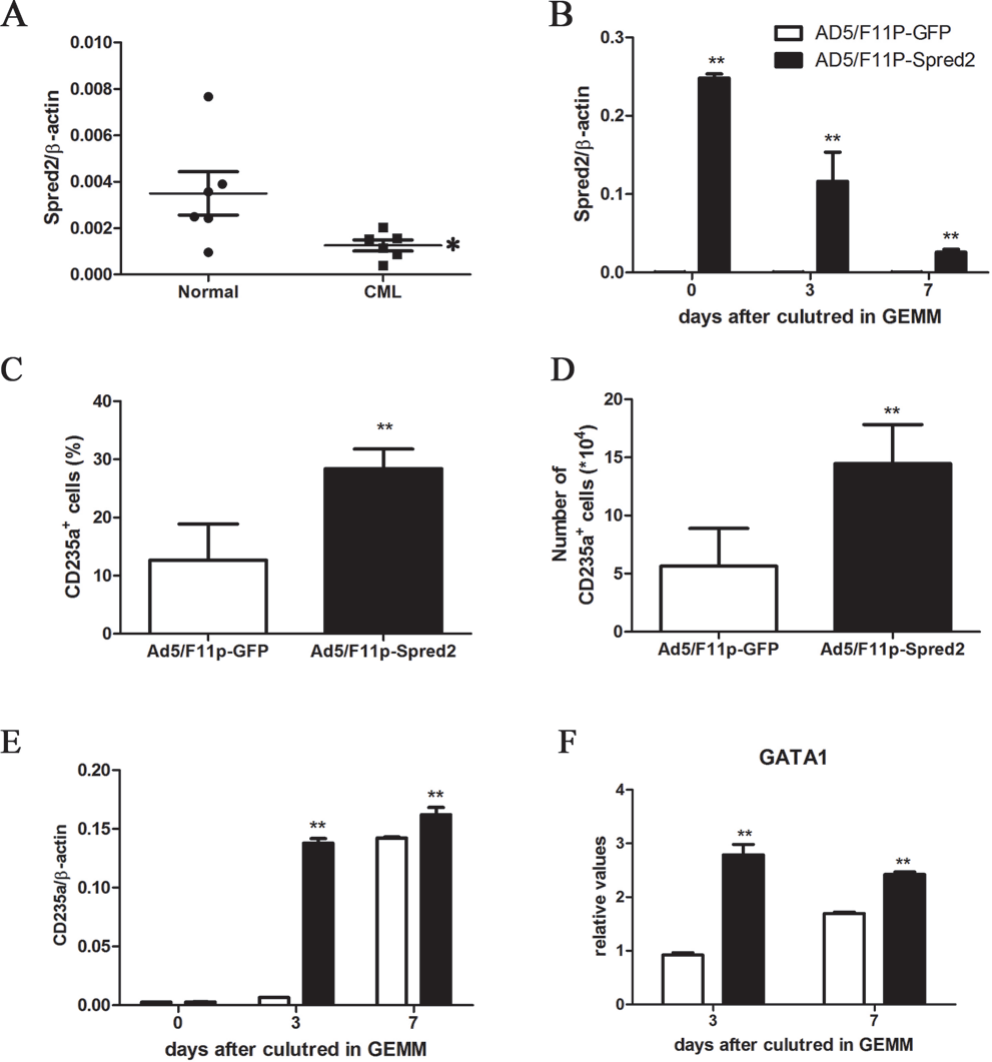
Spred2 induced erythroid differentiation of CML CD34^+^ cells. The mRNA expression of Spred2 in CML patient derived CD34^+^ cells and NBM CD34^+^ cells was detected by using real-time RT-PCR (A). CML CD34^+^ cells were transduced with adenoviral vector Ad5/F11p-Spred2 or Ad5/F11p-GFP at a MOI of 150 and cultured in GEMM medium for 7 days, the expression of Spred2 (B), CD235a (E) and GATA1 (F) was detected by using real-time RT-PCR at day 0, 3 and 7. 2×10^5^ transduced cells were cultured in GEMM medium for 3 days, the percentage of CD235a^+^ cells (C) was detected by flow cytometer and number of generated CD235a^+^ cells were calculated (D). Data are shown as mean±s.d. of three independent experiments. *, p<0.05, vs NBM CD34^+^ cells in A. *, p<0.05; **, p<0.01 vs the Ad5/F11p-GFP group at the same time point in B-F.

Compared to that in NBM CD34^+^ cells, the Spred2 expression was lower notably in CML CD34^+^ cells (Fig. 2A), which was consistent with the impaired differentiation ability of these cells. To clarify the effect of Spred2 restoration on erythroid differentiation of CML cells, the CML CD34^+^ cells were transduced with Ad5/F11p-Spred2, and assayed for erythorid differentiation ability. As shown in Fig 2B, Ad5/F11p-Spred2 transduction resulted in Spred2 restoration in CML CD34^+^ cells cultured in GEMM medium for 7 days. Spred2 restoration increased the generation of CD235a^+^ cells by CML CD34^+^ cells at 3 day after cultured in GEMM system (Fig. 2C–2D). The mRNA expression of CD235a (Fig. 2E) and GATA1 (Fig. 2F) were also upregulated obviously after Ad5/F11p-Spred2 transduction.

### Spred2 was involved in imatinib induced erythroid differentiation of K562 cells

**Fig 3:**
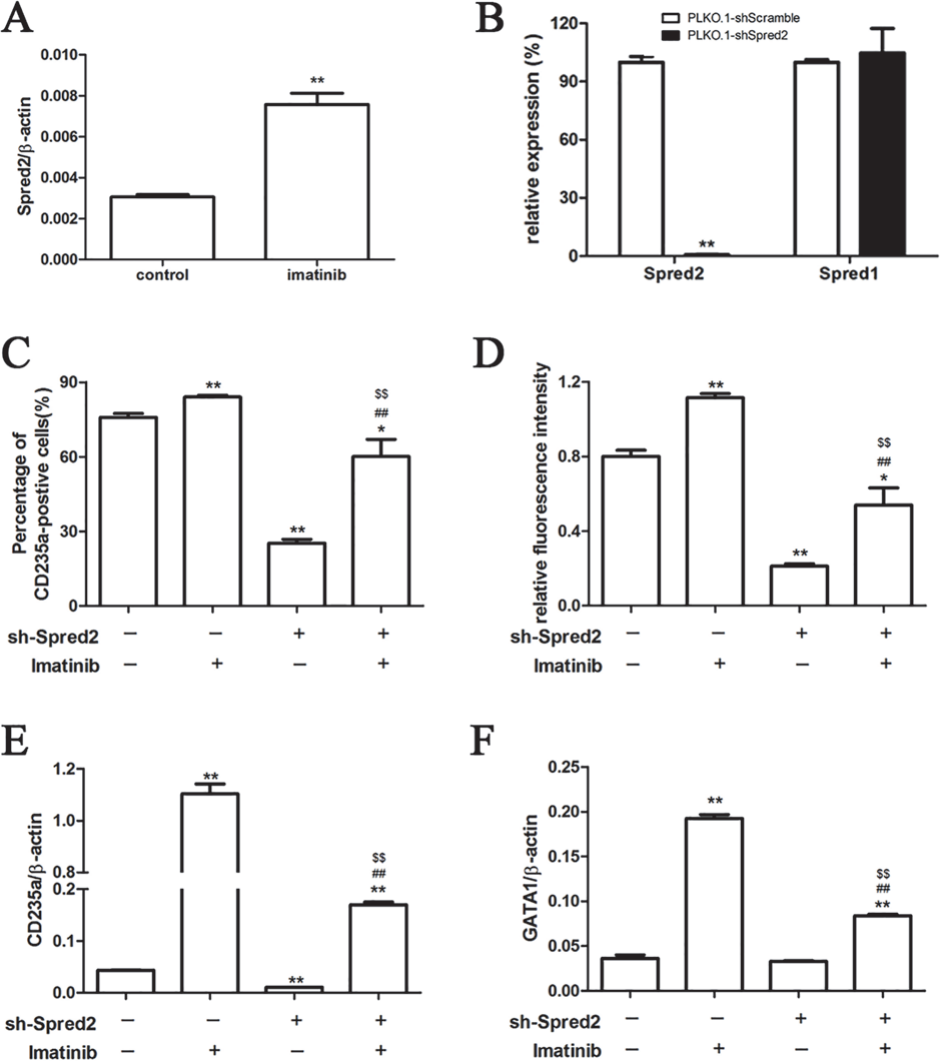
Spred2 was involved in imatinib induced erythroid differentiation. K562 cells were induced by 1 μM imatinib for 48 hours, and the expression of Spred2 was detected by real-time reverse-transcription polymerase chain reaction (RT-PCR) (A). The K562 cells were transduced with PLKO.1-shSpred2 or PLKO.1-shScramble and cultured for 48 hours. The interference efficiency was confirmed by real-time RT-PCR (B). These transduced cells were cultured in the absence or presence of imatinib at a concentration of 1μM for 48 hours, the percentage of CD235a positive cells and relative fluorescence intensity were detected by flow cytometer (C-D), and the mRNA expression of CD235 (E) and GATA1 (F) were also detected by real-time RT-PCR. Data are shown as mean±s.d. of three independent experiments. *, p<0.05,**, p<0.01 vs the first column; #, p<0.05; ##, p<0.01 vs the second column; $, p<0.05,$$, p<0.01 vs the third column.

We investigated the influence of imatinib treatment on Spred2 expression of K562 cells using real-time RT-PCR, and the results showed that Spred2 could be induced by imatinib (Fig. 3A). PLKO.1-shSpred2 transduction inhibited Spred2 expression obviously in K562 cells, while had no effect on Spred1 (Fig. 3B). Interestingly, Spred2 interference could partially block imatinib-induced erythroid differentiation of K562 cells. As shown in Fig. 3C–3D, imatinib treatment increased CD235a expression in K562 cells, whereas Spred2 silence downregulated the expression of CD235a both in presence or absence of imatinib. Furthermore, mRNA expression of CD235a (Fig. 3E) and GATA1 (Fig. 3F) was also downregulated in PLKO.1-sh-Spred2 transduced cells. These results indicated that Spred2 was involved in imatinib induced erythroid differentiation of CML cells.

### Spred2 over-expression enhanced erythroid differentiation induced by imatinib in K562 cells

**Fig 4:**
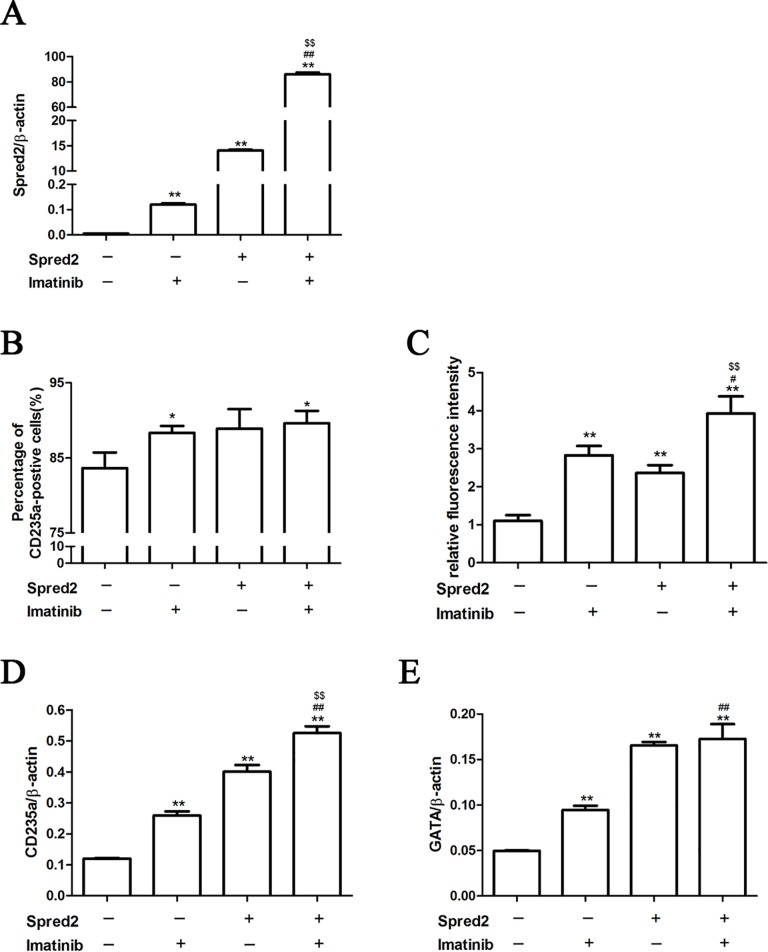
Spred2 over-expression enhanced imatinib-induced erythroid differentiation of K562 cells. K562 cells were infected by adenoviral vector (Ad5/F11p-Spred2 or Ad5/F11p-GFP) at a MOI of 150 and cultured with or without 1μM imatinib for 48 hours. The mRNA expression of Spred2 (A), CD235a (D) and GATA1 (E) were detected by real-time RT-PCR. The percentage of CD235a positive cells (B) and relative fluorescence intensity (C) were detected by flow cytometer. Data are shown as mean±s.d. of three independent experiments. *, p<0.05, **, p<0.01 vs the first column; #, p<0.05; ##, p<0.01 vs the second column; $, p<0.05, $$, p<0.01 vs the third column.

The effects of Spred2 over-expression on erythroid differentiation of K562 cells were also investigated in this study. Ad5/F11p-Spred2 transduction increased Spred2 expression of K562 cells in absence or presence of imatinib (Fig. 4A). Our results showed that imatinib or Spred2 over-expression could increase the CD235a and GATA1 expression, while the combination of imatinib and Spred2 over-expression was much more impressive (Fig. 4B–4E), suggesting the combination might be a potential strategy for CML therapy.

### Spred2 regulated erythroid differentiation through targeting ERK signaling in K562 cells

**Fig 5:**
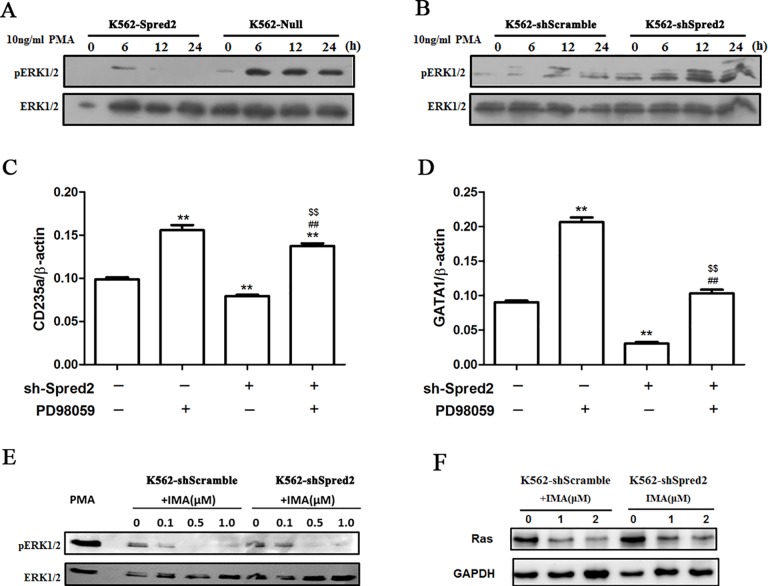
Spred2 mediated erythroid differentiation partly through ERK signalling. Spred2 over-expression cells (K562-Spred2) (A), Spred2 silencing K562 cells (K562-shSpred2) (B) and corresponding control cells (K562-Null and K562-shScramble) were starved deprived of serum overnight and stimulated with 10 ng/ml PMA for the indicated time points. Phosphorylated ERK1/2 and total ERK1/2 were detected by Western blotting. On the other hand, K562-shSpred2 and K562-shScramble cells were treated with imatinib at indicated concentrations for 1 hour, the phosphorylated ERK1/2 and total ERK1/2(E), and Ras (F) were detected. K562 cells were treated by 30μM ERK inhibitor PD98059 1h before lentiviral vector (PLKO.1-sh-Spred2 and PLKO.1-sh-Scramble) transduction. 24h after transduction, the culture medium was replaced by flesh complete culture medium containing 30μM PD98059. Then, the mRNA expression of CD235a and GATA1 were analysed by real-time RT-PCR at 48h post-infection (C and D). Data are shown as mean±s.d. of three independent experiments. *, p<0.05, **, p<0.01 vs the first column; #, p<0.05; ##, p<0.01 vs the second column; $, p<0.05, $$, p<0.01 vs the third column.

Spred2 mediated inhibition of ERK signaling has been reported in K562 cells. In this study, we demonstrated that Spred2 could inhibit PMA induced ERK phosphorylation, while Spred2 interference enhanced PMA induced activation of ERK signaling (Fig. 5A-B). It has been demonstrated above that Spred2 silence could inhibit erythroid differentiation of K562 cells. However, ERK inhibitor, PD98059, partly restore the erythroid differentiation in PLKO.1-shSpred2 transduced K562 cells, suggesting PLKO.1-shSpred2 inhibit erythroid differentiation partly through ERK signaling (Fig. 5C–5D). Furthermore, we also found that imatinib treatment resulted in inactivation of ERK signaling, while Spred2 silence partly restored ERK phosphorylation in imatinib treated K562 cells (Fig. 5E), indicating that imatinib and Spred2 might synergistically inhibit the ERK signaling to regulate erythroid differentiation of K562 cells. Moreover, Spred-2 knockdown also increases Ras expression in K562 cells treated with imatinib, which is consistent to the changes of ERK signals (Fig. 5F).

## Discussion

Spred proteins, a class of selective inhibitors of the Ras-ERK cascade, inhibit cell motility, proliferation, tumor metastasis and Rho-mediated actin reorganization [22–24]. Spred2, a member of Spred proteins, is expressed in the aorta-gonad-mesonephros (AGM) region and functions as a negative regulator in AGM hematopoiesis [25]. In this study, we demonstrated that Spred2 was involved in erythroid differentiation of CML cells induced by imatinib.

Spred2 lies downstream of FoxO3a, which was involved in imatinib-induced cytotoxicity and erythroid differentiation [26–28]. Restored expression of Foxo3a and Spred1 was induced by tyrosine kinase inhibitors, such as imatinib and disatinib [29].Previous reports also showed that Spred2 down-regulation in hematopoietic stem cells of FoxO3a-deficient mice hyper-activated ERK and resulted in hyper-proliferation of neutrophils [30]. We also found the implication of Spred2 in imatinib-induced cell killing of CML cells [17]. However, the role of Spred2 in regulation of erythroid differentiation of CML cells and its mechanisms remain to be fully clarified.

CML is clinically characterized by three phases: an initial chronic phase displaying almost normal myeloid differentiation, followed by an accelerated phase and then the final blast crisis, in which myeloid and lymphoid blasts failed to differentiate and led to abnormal accumulation of immature leukemic blast cells in blood and bone marrow [31]. Our data showed that the expression of Spred2 was down-regulated significantly in CML CD34^+^ cells, and Spred2 over-expression could restore the ability of erythroid differentiation. These data indicated that Spred2 was involved in differentiation of CML cells and might be a candidate target for CML therapy.

Imatinib could induce both cytotoxicity and erythroid differentiation of CML cells [17,32,33]. We further investigated whether Spred2 was involved in imatinib induced erythroid differentiation in CML cells. K562 cell is a bipotent cell line established from a patient in a blast crisis of chronic myeloid leukemia, it possesses variable capacities of differentiation toward erythroid and megakaryocytic cell lineages. We assayed the effects of imatinib and Spred2 on differentiation potential of CML cells. Our results showed that Spred2 over-expression enhanced the erythroid differentiation induced by imatinib, whereras Spred2 silence partly blocked this process. We also demonstrated that imatinib induced Spred2 expression both in primary CML and K562 cells.

Several reports suggested that blockade of BCR-ABL and downstream Ras-ERK pathway by imatinib, geldanamycin, RNA interference of BCR-ABL, herbimycin A, U0126, butyrateand ara-C caused erythroid differentiation of K562 cells [8,34–38]. Others indicated that inhibition of signaling through ERK in K562 cells might be needed to enter the erythroid differentiation process, while the erythroid differentiation after initiation could be enhanced by both activation and inhibition of ERK signaling depending on inducing compound [36]. Based on the previous data that Spred2 inhibited phosphorylated-ERK (p-ERK) in K562 cells, we further demonstrated that Spred2 interference could partly reverse imatinib induced down-regulation of p-ERK level. Our data also showed that MEK-1 inhibitor, PD98059, not only enhanced the erythroid differentiation in K562 cells, but also reversed PLKO.1-sh-Spred2 induced inhibition of erythroid differentiation, indicating that Spred2 interference blocks erythroid differentiation partly through activation of ERK signaling.

## Conclusion

We here demonstrated that Spred2 participated in erythroid differentiation of CML cells. Spred2 was involved in imatinib induced erythroid differentiation partly through inhibition of ERK signaling. These data might provide valuable insights into the mechanisms of differentiation of CML cells and present novel target for developing therapy strategies.
